# Anti-HER-2 engineering antibody ChA21 inhibits growth and induces apoptosis of SK-OV-3 cells

**DOI:** 10.1186/1756-9966-29-23

**Published:** 2010-03-10

**Authors:** AnLi Zhang, Hua Xue, XiaoGuang Ling, Yi Gao, Feng Yang, LianSheng Cheng, Jing Liu, Qiang Wu

**Affiliations:** 1 Department of Pathology, Anhui Medical University, 69# Meishan Road, Hefei, Anhui, 230032, PR China; 2 School of Life Science, University of Science and Technology of China, Hefei, 230027, PR China

## Abstract

**Background and Aims:**

Anti-HER-2 antibodies targeting distinct epitopes have different biological functions on cancer cells. In a previous study, we demonstrated that anti-HER-2 engineering antibody ChA21 was able to bind to subdomain I of HER-2 extracellular domain. In this study, The effects of ChA21 on growth and apoptosis against ovarian carcinoma cell SK-OV-3 over-expressing HER-2 *in vitro *and *in vivo *were investigated.

**Methods:**

Cell growth inhibition was evaluated by MTT assay. Apoptosis was detected by TUNEL stain, transmission electron microscopy and flow cytometry on cultured cells and tissue sections from nude mice xenografts. The apoptosis-related proteins Bax and Bcl-2 were assessed by immunohistochemistry.

**Results:**

We found that treatment of ChA21 caused a dose-dependent decrease of cell proliferation *in vitro *and a significant inhibition of tumor growth *in vivo*. ChA21 therapy led to a significant increase in the induction of apoptosis, and up-regulated the expression of Bax, while the expression of Bcl-2 was down-regulated.

**Conclusion:**

These data suggest that ChA21 inhibits the growth and induces apoptosis of SK-OV-3 via regulating the balance between Bax and Bcl-2.

## Introduction

Ovarian cancer is the most frequent cause of death among all gynecologic cancer patients [1], and there are currently no effective therapeutic approaches for the disease in spite of advances in surgery, chemotherapy, and radiotherapy [2,3]. Hence, the effective treatment for ovarian cancer is urgently needed.

HER-2, also named neu/c-erbB-2, is a key member of the epidermal growth factor receptor (EGFR) family, which comprises an extracellular domain (ECD) with four subdomains (I/L1, II/S1, III/L2, and IV/S2), a single transmembrane domain, and an intracellular tyrosine kinase domain [4,5]. The aberrant activity of HER-2 has been shown to play a key role in the development and growth of tumor cells [6,7]. HER-2 gene over-expressed in ovarian cancer has been reported to be approximately 15-30% [8,9]. HER-2 over-expression in human carcinoma tissues does relate with the poor prognosis but provide the fundamental rationale for the development of immunotherapy to target HER-2. The most attractive humanized antibody against HER-2 is Herceptin [10,11], which blocks HER-2 dimerization and induces apoptosis [12]. It has been used as an agent in first-line treatment of HER-2 over-expressing breast cancer by binding to HER-2 extracellular domain in subdomain IV [13,14]. It was also reported that Herceptin appeared to be a candidate as a treatment modality for HER-2 over-expressing ovarian cancer [15]. ChA21 is an engineered anti-HER-2 antidbody that is prepared by the surface epitope masking (SEM) method, wherein recognized epitopes are mainly located in subdomain I of the HER-2 extracellular domain [16-18].

In previous study, we reported the preparation of an anti-HER-2 monoclonal antibody(MAb) muA21 and found that it could inhibit the growth of the human breast cancer SK-BR-3 cells [19,20]. Subsequently, we cloned the genes of variant regions of this monoclonal antibody, constructed the single-chain Fv (scFv) antibody, and further constructed a chimeric scFv-Fc engineered antibody ChA21 [16]. After that, we constructed a molecular model of Ag-Ab complex based on the crystal structures of the ChA21 scFv and HER-2 ECD, and found that ChA21 recognized epitopes mainly located in subdomain I [18]. In the present study, we hypothesized that ChA21 could bring the similarly effects for the growth inhibition of HER-2 over-expressed SK-OV-3 cells and induction of apoptosis as Herceptin binds to subdomain IV.

## Materials and methods

### Cell line

The HER-2 overexpressing human ovarian cancer cells SK-OV-3 [21] were obtained from the Cell Bank of Shanghai Institutes for Biological Sciences (Shanghai, China). They were cultured in DMEM (Gibco, USA) supplemented with 10% FBS (Gibco, USA) in an incubator with 5% CO_2 _and saturated humidity at 37°C.

### MTT assay

SK-OV-3 (5 × 10^3 ^per well) cells were seeded in 96-well plates and cultured overnight. Then, the medium was replaced with fresh DMEM or the same medium containing ChA21 (prepared as described in previous studies [16,17]) at concentrations of 0.067, 0.2, 0.6, 1.8, 5.4 μg/ml for 72 h, or the cells were treated with ChA21 at the concentration of 5.4 μg/ml for 24, 48, 72, 96 h, respectively. MTT (Sigma, USA) with 20 μl samples was added to each well and incubated for an additional 4 h. Then culture medium was discarded and 150 μl dimethyl sulfoxide (DMSO) was added. OD 570 nm was measured by a multi-well scanning spectrophotometer (Multiskan MK3, Finland). The inhibitory growth rate was calculated as follows: (1 - experimental OD value/control OD value) × 100%.

### Inhibition of ChA21 on SK-OV-3 nude mice xenografts

BALB/c female nude mice (6 weeks old, 18.0 ± 2.0 g) were obtained from Shanghai Laboratory Animal Center (SLAC, China). SK-OV-3 cells (5 × 10^6 ^per mouse) were subcutaneously inoculated into the left flank of the mice. Tumor-bearing mice in which the tumor volume reached about 50 mm^3 ^were selected, and randomized, injected with either sterile normal saline or ChA21(40 mg/kg) twice weekly via caudal vein (i.v) for 5 weeks. Tumor size was measured twice a week and converted to tumor volume (TV) as the following formula: TV (mm^3^) = (a × b^2^)/2, where a and b are the largest and smallest diameters (in millimeters), respectively. All animals were killed after giving ChA21 or sterile normal saline for 5 weeks, and the transplantation tumors were removed, weighed and fixed for further study. The tumor inhibition ratio (TIR) was calculated as follows: (1 - experimental mean weight/control mean weight) × 100% [22].

### Evaluation of potential adverse effects

To evaluate the potential side effects or toxicity on mice during treatment of ChA21, gross measures such as weight loss, ruffling of fur, life span, behavior, and feeding were investigated. The tissue of heart, liver, spleen, lung, kidney, and brain were fixed in 10% neutral buffered formalin solution and embedded in paraffin, and then stained with H&E.

### Transmission electron microscopy

SK-OV-3 cells treated with ChA21 (5.4 μg/ml) for 72 h, as well as 1 mm × 1 mm tumor tissues from nude mice, were fixed with glutaraldehyde and osmium tetroxide. After dehydration in a graded series of acetone and steeping in propyleneoxide, the samples were ultramicrotomed after embedded in Epon 812. The sections were stained with lead citrate, and examined by an electron microscope (JEM-1230, Japan).

### TUNEL staining of apoptotic cells

SK-OV-3 cells (2.5 × 10^4 ^per well) were seeded in 24-well plates with coverslips, and cultured in the medium with 5.4 μg/ml ChA21 for 72 h. Then, the coverslips were taken out, washed, fixed, and stained according to the instruction manual of in situ cell-death detection kits (Roche). The tissue sections from nude mice xenografts were dewaxed and hydrated, and then were incubated with 20 μg/L proteinase K at room temperature for 15 min, followed by incubation with TUNEL reaction mixture. Converter-peroxidase solution was added for further incubation. Labeled nuclei were demonstrated using 3, 3'-diaminobenzidine and counterstained with hematoxylin. Four equal-sized fields were randomly chosen and analyzed, the apoptotic index (AI) was defined as follows: AI (%) = 100 × apoptotic cells/total tumor cells.

### Propidium iodide staining of dead cells for flow cytometry

SK-OV-3 cells were incubated with ChA21 (0.2 or 5.4 μg/ml) for 72 h, harvested and counted, and 1 × 10^6 ^cells were resuspended in 100 μl phosphate-buffered saline (PBS). Then, 5 μl of propidium iodide (PI, Beckman, USA) was added, incubated for 30 min at room temperature in dark. Then the cells were subjected to flow cytometry to measure the death rate (%) with a Beckman Coulter Epics-XL-MCL cytometer (California, USA).

### Immunohistochemical and immunocytochemical staining for Bcl-2 and Bax

The SK-OV-3 cells were cultured and fixed as described above in TUNEL staining. The sections of paraffin-embedded tissue were dewaxed and rehydrated. After inactivating endogenous peroxidase with 3% H_2_0_2_, and blocking cross-reactivity with normal serum, the sections were incubated overnight at 4°C with the Bcl-2 antibody (1:150, Santa Cruz, California, USA) and the Bax antibody (1:150, Santa Cruz, California, USA), respectively. Then, the sections were treated with streptoavidin-peroxidase reagent (Zymed, USA), and the peroxidase label was demonstrated using 3, 3'-diaminobenzidine, counterstained with hematoxylin. Omission of the primary antibody was used as negative control. The immunostained sections were examined by using an Eclipse E800 microscope (Nikon, Japan) coupled to a digital camera. The mean optical density (MOD) of microscopic images was quantitatively analyzed by Image-pro Plus 5.02 (Media Cybernetics Inc, USA).

### Statistical analysis

Data were expressed as mean ± standard deviation ( ± s). Comparison between groups was made by the Independent Samples *t*-test, *P *< 0.05 was considered statistically significant.

## Results

### ChA21 inhibits the growth of SK-OV-3 cells in vitro and in vivo

**Figure 1:**
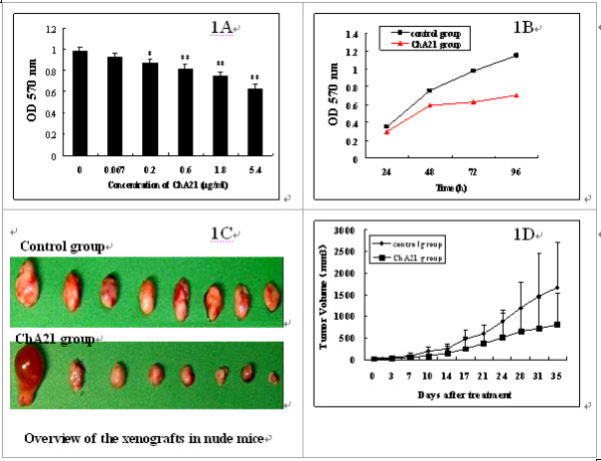
**ChA21 inhibits the growth of SK-OV-3 cells in vitro and in vivo**. (A) Cells were exposed to 0.067-5.4 μg/ml ChA21 for 72 h. (B) Cells were treated with ChA21 at the concentration of 5.4 μg/ml for 24, 48, 72, 96 h, respectively. OD 570 nm was measured by a multi-well scanning spectrophotometer. Significant differences are represented by asterisk (*P *< 0.05) and double asterisk (*P *< 0.01). (C, D) Female BALB/c nude mice were subcutaneously inoculated with human ovarian cancer cells SK-OV-3 (5 × 10^6^) into the left flank of mice. The mice were randomized and injeceted twice weekly via caudal vein with either sterile normal saline or ChA21 (40 mg/kg) for 5 weeks. Tumor size was measured twice a week and converted to tumor volume. ChA21 treatment group have a significantly reduced tumor volume compared with the controls (*P *< 0.05). Results are representative of the mean ± s.e.m. of 8 animals in each group.

To evaluate the effect of ChA21 on cell proliferation, human ovarian cancer cells SK-OV-3 were treated with different doses (0.067-5.4 μg/ml) of ChA21 for 72 h or treated with ChA21 (5.4 μg/ml) for 24, 48, 72, 96 h, respectively. As shown in Fig. 1A, treatment of ChA21 resulted in a dose-dependent inhibition of SK-OV-3 cell proliferation by MTT assay; the growth inhibitory rates were 5.85, 10.92, 16.55, 23.87 and 35.33% at the corresponding concentrations of 0.067, 0.2, 0.6, 1.8 and 5.4 μg/ml, respectively. As shown in Fig. 1B, treatment of ChA21 also resulted in a time-dependent inhibition of SK-OV-3 cells, the growth inhibitory rates were 14.78, 22.89, 34.43 and 39.85% at the corresponding times of 24, 48, 72, 96 h.

Female BALB/c nude mice were subcutaneously inoculated with human ovarian cancer cells SK-OV-3 (5 × 10^6^) into the left flank of mice. The mice were randomized and injected twice weekly via caudal vein with either sterile normal saline or ChA21 (40 mg/kg) for 5 weeks. As shown in Fig. 1C, D, the tumor volume (mm^3^) in the control group grew remarkably fast, reaching 1664.5 ± 1028.7 after 35 days injection. In contrast, the tumor volume (mm^3^) of mice treated with ChA21 was significantly (*P *< 0.05) smaller than the controls, reaching only 813.6 ± 724.8. The mean weight (g) of the transplantation tumors in ChA21 treatment group was 0.78 ± 1.14, which also significantly (*P *< 0.05) decreased as compared to that in the controls (1.24 ± 0.94). In addition, the tumor inhibition ratio reached 37.1%.

### Observation of Potential Toxicity

To evaluate the possible adverse effects of the treatments, weight of mice was monitored every 3 days throughout the whole experiment and considered a variable for evaluation of systemic well-being or cachexia. No significant differences in weights were found between the two groups. No adverse consequences in other gross measures such as ruffling of fur, behavior, feeding, or toxic death were observed. In the histopathological examination of the heart, liver, spleen, lung, kidney and brain, no significant injuries were found after 5 weeks injection (data not shown).

### ChA21 induces apoptosis of SK-OV-3 cells in vitro and in vivo

**Figure 2:**
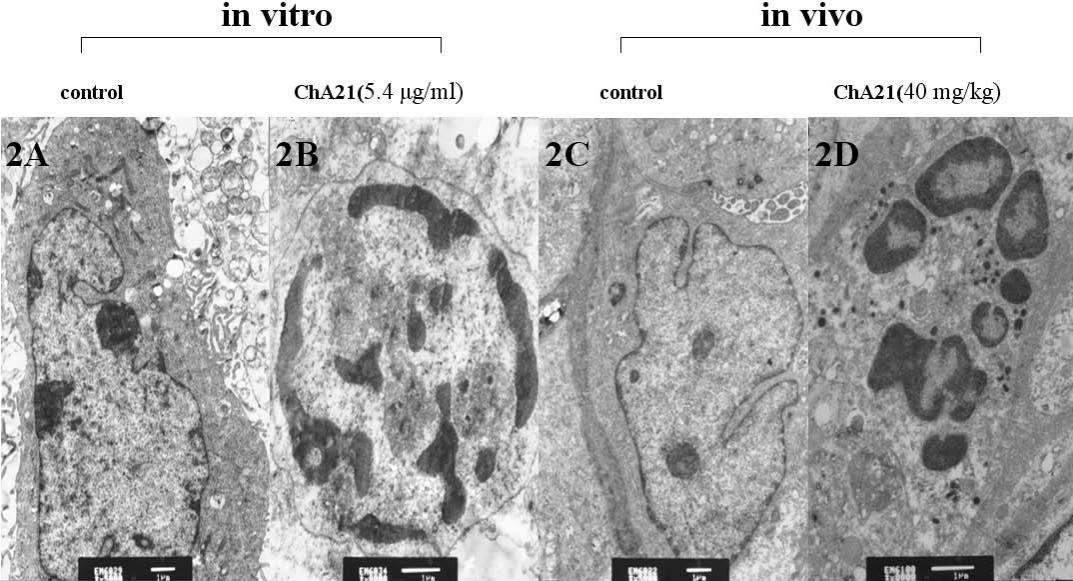
**Transmission electron microscopy observation**. After ChA21 (5.4 μg/ml) treatment for 72 h or the tumor tissues removed from nude mice treated ChA21 (40 mg/kg) for 5 weeks, a large number of cells presented a series of ultrastructural changes of apoptosis (B, D). On the contrary, control cells were morphologically normal and exhibited no signals of apoptosis (A, C). (magnification: A, C × 3000; B, D × 8000).

Using transmission electron microscopy, we discerned the ultrastructural changes of SK-OV-3 cells induced by ChA21. After treatment of ChA21 (5.4 μg/ml) for 72 h or the tumor tissues removed from nude mice treated ChA21 (40 mg/kg) for 5 weeks, a large number of cells presented a series of ultrastructural changes such as chromatin condensation, chromatin crescent, and nucleus fragmentation (Fig. 2B, D), all of which were characteristics of cells undergoing apoptosis. On the contrary, control cells were morphologically normal and exhibited no signals of apoptosis (Fig. 2A, C).

**Figure 3:**
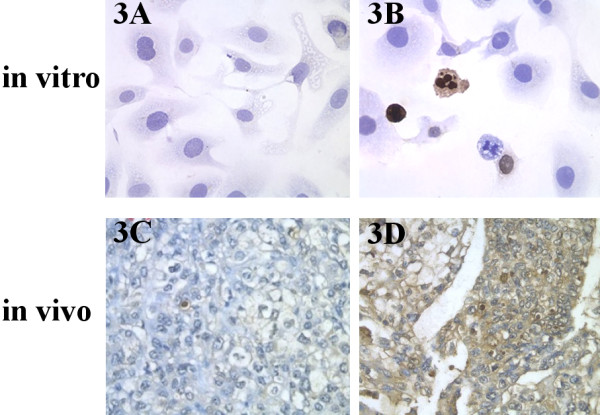
**ChA21 induces apoptosis of SK-OV-3 cells in vitro and in vivo by TUNEL staining**. (A): Control group in vitro (B): ChA21 (5.4 μg/ml) group in vitro (C): Control group in vivo (D): ChA21 (40 mg/kg) group in vivo. Cells cultured with coverslips and tissue sections were stained with the TUNEL agent and examined by light microscopy. Less apoptotic cells were detected in control group, whereas more apoptotic cells were detected in ChA21 treatment group. (magnification: × 200)

Cells cultured on coverslips and tissue sections from the above experiments were stained with the TUNEL agent, and examined by microscopy. Less apoptotic cells were detected in the control group, whereas more apoptotic cells were detected in ChA21 treatment group (Fig. 3). The apoptotic cells on coverslips and tissue sections were counted to calculate the apoptotic index. In vitro, the AI value in ChA21 (5.4 μg/ml) treatment group reached 16.22 ± 1.05, which was higher than that in the controls (6.22 ± 1.09, *P *< 0.05). In vivo, the AI value in ChA21 (40 mg/kg) treatment group reached 9.16 ± 2.44, which was also higher than that in the controls (3.45 ± 0.98, *P *< 0.05).

**Figure 4:**
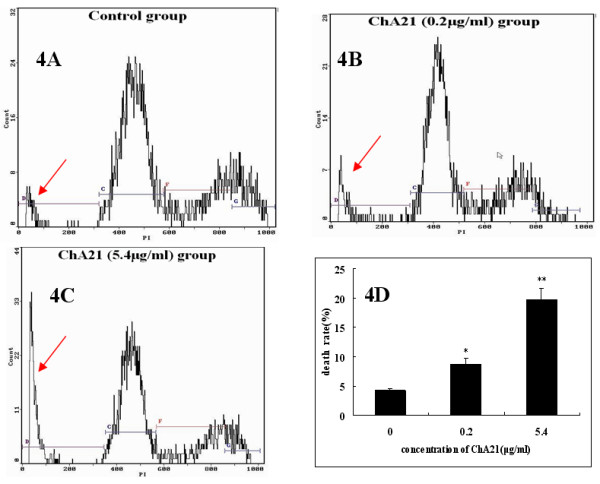
**ChA21 induces death of SK-OV-3 cells in vitro with PI staining**. SK-OV-3 cells were incubated with ChA21 (0.2 or 5.4 μg/ml) for 72 h, and flow cytometric analysis was used to measure the death rate. Significant differences in death rates are represented by asterisk (*P *< 0.05) and double asterisk (*P *< 0.01).

SK-OV-3 cells were incubated with ChA21 (0.2 or 5.4 μg/ml) for 72 h, and flow cytometric analysis was used to measure the death rate. As shown in Fig. 4, there was a significant difference between ChA21 group and control group in the death rate (%) (*P *< 0.05). After the treatment of SK-OV-3 cells with ChA21 (0.2 or 5.4 μg/ml) for 72 h, the death rate (%) reached 8.75 ± 0.97, and 19.73 ± 1.99, respectively.

### Expression of Bcl-2 and Bax

**Figure 5:**
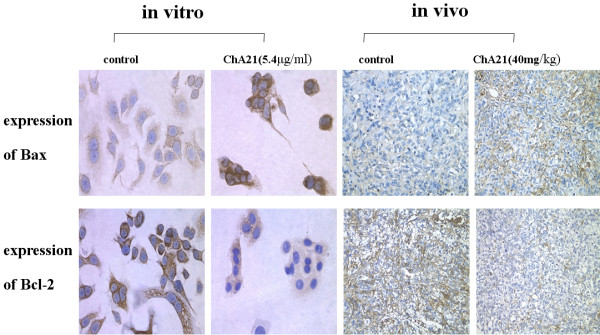
**Expression of Bcl-2 and Bax as detected by immunohistochemistry**. Detection of the expression of apoptosis-related proteins of Bcl-2 and Bax showed that ChA21 therapy could upregulate the expression of Bax and downregulate the expression of Bcl-2 in vitro and in vivo.

**Figure 6:**
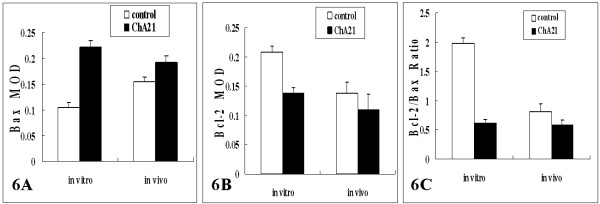
**The MOD values on expression of Bcl-2 and Bax**. MOD values of Bax in ChA21 treatment group were higher than those in the control group (*P *< 0.05), while MOD values of Bcl-2 and the ratio of Bcl-2 to Bax were lower (*P *< 0.05) both in vitro and in vivo. (magnification: in vitro × 400; in vivo × 200).

Detection of the expression of apoptosis-related proteins of Bcl-2 and Bax by immunohistochemistry showed that ChA21 therapy could up-regulate the expression of Bax, and down-regulate the expression of Bcl-2 (Fig. 5), thereby reducing the ratio of Bcl-2/Bax *in vitro *and *in vivo*. As shown in Fig. 6, MOD values of Bax in ChA21 group were higher than those in control group (*P *< 0.05), while MOD values of Bcl-2 and the ratio of Bcl-2 to Bax were lower (*P *< 0.05) both *in vitro *and *in vivo*.

## Discussion

In recent years, a number of monoclonal antibodies (MAb) have been developed against HER-2 ECD, such as 4D5 (Herceptin, trastuzumab) and 2C4 (Pertuzumab) [10,23]. Herceptin is a humanized recombinant MAb that was first approved by the U.S. FDA for use in HER-2 over-expressing metastatic breast cancer. Current studies show that it appears to be a candidate as a treatment modality for HER-2 over-expressing ovarian cancer as well [24]. However, more studies in clinical application showed that there is an increased incidence of serious cardiac events, particularly when Herceptin was administered in combination with anthracyclines [25,26], and pulmonary complications also had been reported [27]. Patients who have had a significant therapeutic effect for a time by Herceptin treatment started to appear the drug resistant [28,29]. Moreover, according to the surveyed data about the clinical therapeutic effect of Herceptin, the therapeutic effective rate of Herceptin treated alone to patients with HER-2 over-expressed only reached 12-14% [30]. These results urge people to conduct more researches, regarding the mechanism of antibodies curing the neoplasms, and develop novel humanized recombinant MAb for HER-2.

Therefore, three strains of murine MAb A18, A21, and A22, which direct against HER-2 ECD were developed, and MAb A21 was found to specifically inhibit the growth of HER-2 over-expressing cells [20]. To reduce the potential for generating a human anti-mouse immune response, Murine MAb A21 was humanized to develop an anti-HER-2 engineering antidbody, ChA21 [16,17]. In previous study, we constructed a molecular model of Ag-Ab complex based on the crystal structures of the ChA21 scFv and HER-2 ECD [18]. Unlike Herceptin that binds to subdomain IV, ChA21 recognizes epitopes that are mainly located in subdomain I. It is possible, that anti-HER-2 antibodies targeting distinct epitopes have different biological functions on cancer cells with different mechanisms [31].

Thus, in the present study, we confirmed that ChA21 binding to subdomain I could inhibit the growth and induce apoptosis of HER-2 over-expressing human ovarian cancer cells SK-OV-3 *in vitro *and *in vivo*. The results showed that *in vitro*, the cell growth was significantly inhibited by ChA21 in a dose- and time-dependent manner. Likewise, ChA21 (40 mg/kg) inhibited the growth of SK-OV-3 cells *in vivo *with the tumor inhibition ratio of 37.1%. It is known that apoptosis is the programmed death of cells, a variety of studies have revealed that the uncontrolled growth of neoplasms is not only the cause of the over growth but also the loss of natural apoptosis [32,33]. Therefore, the antibody that is capable of inducing cancer cells apoptosis would be helpful for cancer treatment. In this study, transmission electron microscope, TUNEL staining and flow cytometry were used to detect apoptosis, and the results demonstrated that ChA21 could induce apoptosis on SK-OV-3 cells both *in vitro *and *in vivo*. Hence, we can deduce that the growth inhibition of ChA21 on SK-OV-3 cells was at least partially contributed by its role of apoptosis induction. To further investigate the possible molecular mechanism of apoptosis induced by ChA21, apoptosis-regulated proteins Bcl-2 and Bax were detected by immunocytochemistry and immunohistochemistry. It is known that Bcl-2 gene acts to inhibit apoptosis, while Bax gene induces apoptosis. The imbalanced expression of Bcl-2 to Bax protein influences the apoptosis of cells stimulated by either external or internal factors [34,35]. Recent studies reported that HER-2 over-expression is accompanied by up-regulation of Bcl-2 and down-regulation of Bax [36,37]. Our results showed that after exposure to ChA21, Bcl-2 expression of SK-OV-3 cells was decreased, and Bax expression was increased, resulting in a decrease in Bcl-2/Bax value. Therefore, we concluded that one of the pathways of ChA21 inducing apoptosis might up-regulate Bax expression, and down-regulate Bcl-2 expression.

In conclusion, the results indicate that ChA21 could inhibit growth and induce apoptosis of human ovarian cancer cell line SK-OV-3 via regulating the balance between Bax and Bcl-2. It suggests that ChA21 might be a new promising candidate in the treatment of HER-2 over-expressed ovarian cancers. In addition, the mechanisms of ChA21 inhibits SK-OV-3 cells growth not only via inducing apoptosis, but also by interfering with HER-2 heterodimerization and affecting HER-2 signaling pathway, and further study is needed.

## Competing interests

The authors declare that they have no competing interests.

## Authors' contributions

ZAL carried out the animal experiment, XH carried out the cells experiment, WQ participated in the design of the study. LXG carried out the transmission electron microscopy observation. YF carried out the immunohistochemical staining. YG participated in the study design. CL carried out the data collection. LJ carried out the design of the study. All authors read and approved the final manuscript.
